# Infant Low Birth Weight Prediction Using Graph Embedding Features

**DOI:** 10.3390/ijerph20021317

**Published:** 2023-01-11

**Authors:** Wasif Khan, Nazar Zaki, Amir Ahmad, Jiang Bian, Luqman Ali, Mohammad Mehedy Masud, Nadirah Ghenimi, Luai A. Ahmed

**Affiliations:** 1 Department of Computer Science and Software Engineering, College of Information Technology, United Arab Emirates University, Al Ain P.O. Box 15551, United Arab Emirates; 2 Department of Information Systems and Security, College of Information Technology, United Arab Emirates University, Al Ain P.O. Box 15551, United Arab Emirates; 3 Department of Health Outcomes and Biomedical Informatics, College of Medicine, University of Florida, Gainesville, USA; 4 Department Family Medicine, College of Medicine and Health Sciences, United Arab Emirates University, Al Ain P.O. Box 15551, United Arab Emirates; 5 Institute of Public Health, College of Medicine and Health Sciences, United Arab Emirates University, Al Ain P.O. Box 15551, United Arab Emirates; 6 Zayed Centre for Health Sciences, United Arab Emirates University, Al Ain P.O. Box 15551, United Arab Emirates

**Keywords:** low birth weight, knowledge graph, topological features, healthcare, birth weight prediction

## Abstract

Low Birth weight (LBW) infants pose a serious public health concern worldwide in both the short and long term for infants and their mothers. Infant weight prediction prior to birth can help to identify risk factors and reduce the risk of infant morbidity and mortality. Although many Machine Learning (ML) algorithms have been proposed for LBW prediction using maternal features and produced considerable model performance, their performance needs to be improved so that they can be adapted in real-world clinical settings. Existing algorithms used for LBW classification often fail to capture structural information from the tabular dataset of patients with different complications. Therefore, to improve the LBW classification performance, we propose a solution by transforming the tabular data into a knowledge graph with the aim that patients from the same class (normal or LBW) exhibit similar patterns in the graphs. To achieve this, several features related to each node are extracted such as node embedding using node2vec algorithm, node degree, node similarity, nearest neighbors, etc. Our method is evaluated on a real-life dataset obtained from a large cohort study in the United Arab Emirates which contains data from 3453 patients. Multiple experiments were performed using the seven most commonly used ML models on the original dataset, graph features, and a combination of features, respectively. Experimental results show that our proposed method achieved the best performance with an area under the curve of 0.834 which is over 6% improvement compared to using the original risk factors without transforming them into knowledge graphs. Furthermore, we provide the clinical relevance of the proposed model that are important for the model to be adapted in clinical settings.

## 1. Introduction

Infant birth weight (BW) is an important factor that must be considered during the clinical evaluation of newborns. Infants born with a BW < 2500 g are considered as having a low birth weight (LBW) [1]. Several contributing factors are associated with LBW, such as low pregnancy weight, low maternal caloric intake, short stature, prematurity, smoking, and female sex of the infant [1]. Infants with LBW are at a higher risk of adverse health outcomes such as intellectual disabilities, learning disabilities, lower IQ, hearing and visual disabilities, obesity, diabetes, and long-term disabilities including premature death [2,3,4]. Every year, nearly 20 million babies, nearly 14.6% of births worldwide, are born with LBW [5,6]. This is an alarming concern; therefore, timely identification of LBW fetuses is essential to minimize the risk of LBW by taking appropriate clinical interventions. 

In sectors such as healthcare, the utmost focus is given to introducing key enablers that aid prolonged health through proper care and treatment. To achieve this, machine learning (ML)-based algorithms have demonstrated promising performance to aid medical personnel in making informed decisions. Table 1 shows a brief overview of several studies conducted to predict LBW using several ML models by utilizing maternal risk factors associated with LBW. For example, Faruk et al. [7] proposed an LBW prediction model using multiple ML models by utilizing data from 12,055 women, noting eight risk factors for each patient. The authors showed that the random forest (RF) method achieved the best performance. Feng et al. [8] used binary support vector machine (SVM) classification to predict fetal weight using ultrasound features from a dataset of 7875 obtained from a hospital in China. Lu et al. [9] proposed an ensemble learning model comprising three models: RF, XGBoost, and LightGBM. The ensemble learning model was based on a genetic algorithm (GA) and was applied to estimate the fetal weight at any gestational age. The authors used a dataset of 4214 women with 14 features obtained from a hospital in China and showed that the proposed ensemble model with a GA achieved better performance than the individual models. Trujillo et al. [10] used data from 250 women with 23 features obtained from a healthcare center in Mexico for infant BW prediction using the support vector regression (SVR) algorithm. Pollob et al. [11] recently built an LBW classification model using ML on a dataset of 2351 instances with 17 risk factors obtained from Bangladesh. The authors demonstrated that logistic regression (LR) achieved the best classification performance. Do et al. [12] used ML to predict mortality in very LBW infants from a dataset of 7472 infants obtained from different hospitals in Korea, and showed that artificial neural networks (ANN) achieved the best performance in predicting mortality in these infants. Lin et al. [13] used ML models to predict the hospital stay of very LBW infants from a dataset of 3519 infants obtained from hospitals in Taiwan.

Several studies concerning infant birth weight among the population of the United Arab Emirates have been carried out. Abdulrazzaq et al. [14] conducted a study to determine the incidence of LBW from data of 3514 births obtained from three hospitals in Al Ain in 1991. The authors showed that, in the selected population, the rate of LBW was 8.4%, and the important risk factors identified were multiple pregnancies, premature membrane rupture, and previous pregnancies with LBW. Nasir et al. [15] used statistical modeling to identify the prevalence and risk factors associated with LBW in a hospital in Ajman, using data from 197 pregnant women between January 2011 and December 2012. The authors showed that 80.7% of LBW cases were observed in women aged—20–34 years, of which 62% were non-Arabs. Another study conducted by Nasir et al. [16] showed that the important risk factors associated with LBW infants were light weight, short interpregnancy interval, nulliparity, and first-cousin marriage. Detailed work related to infant BW estimation and classification can be found in [2,17]. 

Although many studies have been conducted on LBW classification (Table 1), the performance of these studies can be improved. The algorithms used in these studies ignore the relationships between different entities and rely on grid-like data [18,19]. Furthermore, the core assumption of ML algorithms is that the patients in a dataset are independent of each other [18]. However, patients might have relationships that share the same diseases or complications [20,21]. Thus, for ML algorithms, extraction of useful information from complex data with relational structures is challenging [22]. 

To address this issue, we propose a novel solution that incorporates node embeddings and graph topological features extracted from the knowledge graph (KG) for infant BW prediction. We achieve this by first transforming the original dataset into a graph and then extracting useful graph features to construct a feature vector for prediction. KG-based solutions are promising solutions that have shown tremendous performance in many applications, including healthcare [18,19,21,22,23,24,25,26,27,28]. However, KG-based solutions are limited to graph datasets, and few studies have focused on extracting graph features from real-life tabular datasets, especially for infant BW prediction. This study addresses these limitations. The contributions of this study are as follows:

1. A well-curated dataset obtained from 3453 patients with 41 important risk factors was used for infant BW prediction in the UAE.
2. Experiments were performed using the five most commonly used ML classifiers on the original tabular dataset and graphs obtained from the original dataset.
3. A detailed performance evaluation was performed using the original risk factors, graph features, and combinations of these features.

The remainder of this paper is organized as follows: Section 2 describes the materials and methods used to explain the proposed methodology. Section 3 explains the experimental results, followed by a discussion of the results in Section 4. Finally, we provide the conclusions and discuss future work in Section 5.

## 2. Materials and Methods

**Figure 1:**
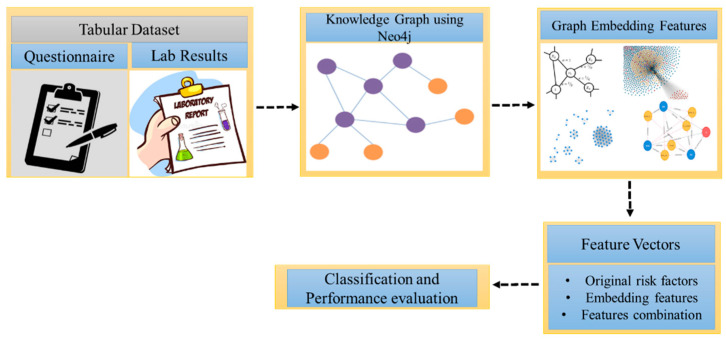
Proposed methodology.

An overview of the proposed method, which consists of several modules, is shown in Figure 1. First, we explain the dataset collection and data preprocessing, followed by the transformation of the tabular dataset into a knowledge graph. We extract several useful graph features (node embeddings and graphs features), followed by feature vector creation. Finally, we evaluate the performance of multiple ML models using different performance metrics. Each module is described in the following subsections.

### 2.1. Dataset Collection and Data Preprocessing

The dataset used in this study is obtained from a currently ongoing prospective maternal and child cohort study in Al Ain, UAE [29]. Data on birth weight and other 41 potential risk factors selected based on literature [2] and medical justification were obtained using self-administered questionnaires answered by the pregnant women during pregnancy or retrieved from their medical records. LBW was defined as an infant whose weight was less than 2500 g.

The study was approved by the Abu Dhabi Health Research and Technology Ethics Committee (DOH/CVDC/2022/72). Informed written consent was obtained from the participant prior to the data collection. The details regarding the study can be found in [29]. The descriptive statistics of the original risk factors used in this study are provided in the Supplementary Table S1. 

### 2.2. Problem Formulation and Knowledge Graph Construction

The dataset ($D_{1}$), as explained in Section 2.1, is transformed into a graph. $D_{1}$ consists of 3453 patients with 37 risk factors. The first task is to transform $D_{1}$ into a graph G that consists of nodes V and edges (links) E. The edges are connected to a pair of nodes, represented as G=(V,E) where $V=\left\{v_{1},v_{2},v_{3},\ldots \ldots v_{n}\right\}$ and $E=\left\{\left(v_{i},v_{j}\right)\;for\;v_{i},v_{j}\in V\right\}$. Each patient and disease are represented as a node, whereas the link between them is represented by an edge. Based on [21], we assume that patients with similar complications will have stronger relations/edges.

The KG was constructed by identifying nodes, properties, and edges. Each patient was considered as a node, while factors including age, body mass index (BMI), and height were the node properties associated with each patient. Diseases and complications were also considered nodes that were connected using an edge with the associated patients. For instance, if a patient had gestational diabetes, there would be an edge between the patient and the gestational diabetes node. After transforming the tabular data into a KG, we extracted several graph features, as explained below.

#### 2.2.1. Node Embeddings

**Figure 2:**
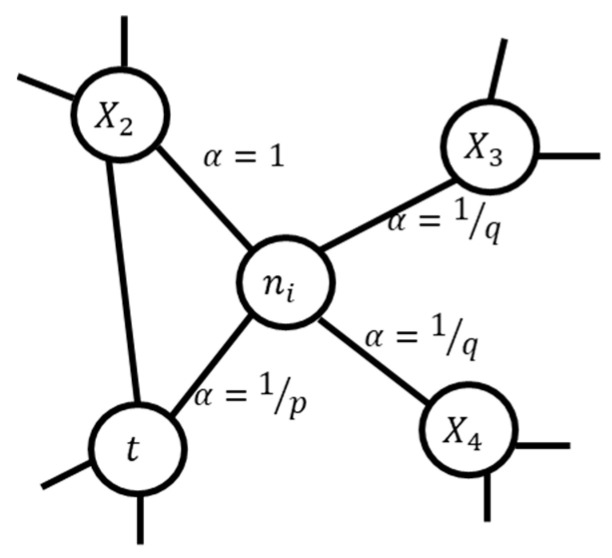
Illustration of the Node2Vec node embedding algorithm [30].

The node embedding algorithm maps the graph structure and relationships into a set of vectors while preserving its structural information. Node2Vec, proposed by Grover and Leskovec [30], is a scalable node embedding algorithm that can efficiently learn the continuous representation of nodes in a graph. Node2Vec uses a flexible biased random walk that explores neighboring nodes using both depth-first search (DFS) and breadth-first search (BFS) strategies. Consider the sample graph in Figure 2 which shows the currently visited node (n) using the rank walk transitioned from t. The next node after n must be determined based on the transition probabilities $\pi_{nx}$ on the edges (n,x). In the Node2Vec algorithm, 2nd order random walk is used, which is based on two parameters p and q where the unnormalized transition probability is used. $\pi_{nx}=\alpha_{pq}\left(t,x\right)\cdot w_{nx}$. where $w_{nx}$ is the static edge weight and $\alpha_{pq}\left(t,x\right)$ can be represented as:

(1) αpq(t,x)={1p if dtx=01 if dtx=11q if dtx=2

$d_{tx}$ represents the shortest distance between nodes t and x which can be in either {0,1,2} to guide the walk. p is a return parameter that controls the likelihood of revisiting a node while walking, while q is the in-out parameter that controls the inward and outward nodes. For instance, a higher value of q will follow BFS behavior, that is, it will visit nodes locally, whereas a lower value of q will enforce the walk to visit farther nodes, thereby approximating DFS behavior. Further details of the Node2Vec algorithm can be found in [29].

#### 2.2.2. Graph Topological Features

In addition to node embedding, we extract multiple graph topological features such that we obtain a feature vector $f=\left\{f_{1},f_{2},\;f_{3},\;\ldots f_{n}\right\}\;$ which is given to ML models for classification. Descriptions of the extracted graph topological features are briefly explained below:

- Graph degree;

We calculate the node degree, which is defined as the number of edges connected to a node $n_{i}$ in a directed graph represented by $d\left(n_{i}\right)=2\overset{n}{\underset{j}{\sum }}n_{i↔j}$, where $d\left(n_{i}\right)$ is the degree d of a node $n_{i}$ in a given graph G. The term $n_{i↔j}$ represents the edge of $n_{i}$ with its adjacent node $n_{j}$. A node degree consists of both in-degree and out-degree, which is the number of edges coming to a node $n_{i}$ represented by $d^{-}\left(n_{i}\right)=\overset{n}{\underset{j}{\sum }}d\left(n_{i\leftarrow j}\right)$ and the number of edges coming out from a node is represented by $d^{+}\left(n_{i}\right)=\overset{n}{\underset{i}{\sum }}d\left(n_{i\to j}\right)$, respectively. A weighted degree is the sum of the in-degree and out-degree, represented as $d^{weighted}\left(n_{i}\right)=d^{-}\left(n_{i}\right)+d^{+}\left(n_{i}\right)$;

- Closeness centrality;

The closeness centrality is the distance measured by a node to reach other nodes in $G_{i}$. If the node $n_{i}$ has the shortest path with other nodes in $G_{i}$, then the CC is higher [31], as represented as CC(ni)=1∑j=1nd(ni,nj) for i≠j;

- Betweenness centrality;

Betweenness centrality is defined as the importance of a node being in between other nodes, that is, the shortest path a node $n_{i}$ has with other nodes [32]. It is represented as $BC\left(n_{i}\right)=\underset{i\neq j\neq k}{\sum }\frac{\sigma_{i,j}\left(n_{i}\right)}{\sigma_{i,j}}$ where $\sigma_{i,j}$ is the shortest path between any two nodes in $G_{i}$;

- Eigenvector centrality;

This measures the importance of a node while considering the importance of its neighbors. A node is considered influential if the eigenvector centrality (EC) of the node and its neighbor is higher [33]. The EC for node $n_{i}$ can be represented by $n_{i}=\frac{1}{\lambda }\overset{n}{\underset{j}{\sum }}a_{i,j}.n_{i}$;

- Hub;

The hub represents the nodes connected to many other nodes in graph G. Because hub nodes are highly authoritative, with a large number of neighbors, they are widely used in many applications, including outbreak detection, page search, and network analysis [34];

- Authority;

Authority is the amount of information a node holds by connecting to many good hubs [19,35]. For node $n_{i}$ the authority can be represented as $n_{i}=\underset{j\to i}{\sum }n_{j}$ where j→i indicates that there is a link from j to i. Conversely, a good hub, as represented by whether it is connected with good authorities, is represented as $n_{i}=\underset{i\to j}{\sum }n_{j}$;

- PageRank;

The PageRank (PR) algorithm [36] ranks the importance of a node for a graph G. The PR for a node $n_{i}$ can be represented as $PR\left(n_{i}\right)=\frac{1-c}{n}+c.\underset{j}{\sum }\frac{PR\left(n_{j}\right)}{d^{+}\left(n_{i}\right)},$ where c is the dumping factor usually maintained around 0.85 (between 0 and 1) [36];

- Clustering coefficient;

This shows the probability that a node $n_{i}$ has two connected neighbors. Mathematically, the ratio of triangles to the ratio of triples node $n_{i}$ in a graph G is represented as Cc(ni)=λ(ni)d(ni)2−d(ni)2;

- K-nearest neighbors;

This returns the nearest neighbors of any node $n_{i}$ by calculating the Euclidean distance to its neighbor nodes $n_{j}$ for i≠j;

- Node similarity;

This compares similar nodes based on their neighbors. Nodes are considered similar if they have the same neighbors. Computing node similarity between two nodes $n_{1}$ and $n_{2}$ using Jaccard similarity can be calculated as $J\left(n_{1},n_{2}\right)=\frac{\left|n_{1}\cap \;n_{2}\right|}{\left|n_{1}\cup \;n_{2}\right|}$;

- Community detection;

This metric identifies communities in the graph. We have used the Louvain algorithm [37], which is fast and scalable. 

#### 2.2.3. Feature Combination for Classification

Feature combination is an effective method for better classification [38]; therefore, to improve the classification performance, we combine graph embedding features with the original risk. Overall, we aim to perform experiments by utilizing the original risk factors, graph-based features, and a combination of features using multiple ML classifiers. 

#### 2.2.4. Machine Learning Models

To evaluate the performance of our proposed method, we aim to use multiple ML models such as random forest (RF) [39], support vector machine (SVM) [40], logistic regression (LR) [41], naïve Bayes (NB) [42], multi-layer perceptron (MLP) [43], XGBoost [44], and LightGBM [45]. The parameters used for these ML models are shown in Table 2.

#### 2.2.5. Performance Metrics

We use multiple performance metrics to better evaluate the performance of the method, such as the weighted average of precision, recall, and F-score [46]. Since our dataset is class imbalance, therefore, we also used the area under the curve of sensitivity versus false positive rate (AUC-ROC), and precision–recall (PR) value [2,43], which are represented in the equations below:

(2) Precision=TPTP+FP

(3) Recall=TPTP+FN

where TP, FP, and FN are true positives, false positives, and false negatives, respectively.

Meanwhile, the F-score can be calculated using the equation below:

(4) $$\mathrm{Fscore}=2\frac{\mathrm{Precision}*\mathrm{Recall}}{\mathrm{Precision}+\mathrm{Recall}}$$

The PR values will be calculated using:

(5) $$y=\frac{TP_{A}+x}{TP_{A}+x+FP_{A}+\frac{FP_{B}-FP_{A}}{TP_{B}-TP_{A}}.x}$$

where y is the precision value between two points A and B at a particular point $TP_{A}+x$, where $x=\left[1,\;TP_{B}-TP_{A}\right]$. The details regarding the PR value can be found in [47].

## 3. Experiments and Results

A dataset of 3453 pregnant women was used in the experiments. In addition, 3062 (11.32%) pregnant women gave birth to normal BW infants while 391 delivered LBW infants. The mean (standard deviation, SD) maternal age was 31.6 (6.07) years. More descriptive statistics of the sociodemographic and clinical characteristics of pregnant women are shown in the Supplementary Table S1. Experiments were repeated five times using a five-fold cross-validation technique, and the mean and SD results are represented in Table 3, Table 4, Table 5, Table 6, Table 7, Table 8 and Table 9. Experiments were performed on the original risk factors, graph topological features, and a combination of features. The knowledge graph was constructed using Neo4j [44,45], and graph algorithms were implemented in Neo4j Graph Data Science and Py2Neo. For classification, experiments were performed using Python 3.8. 

**Figure 3:**
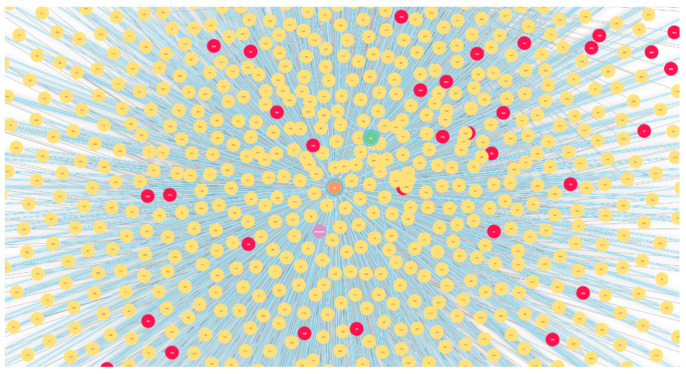
An example of the knowledge graph constructed from the tabular data. Red nodes represent relatively older patients whose age is higher than 45 years.

**Figure 4:**
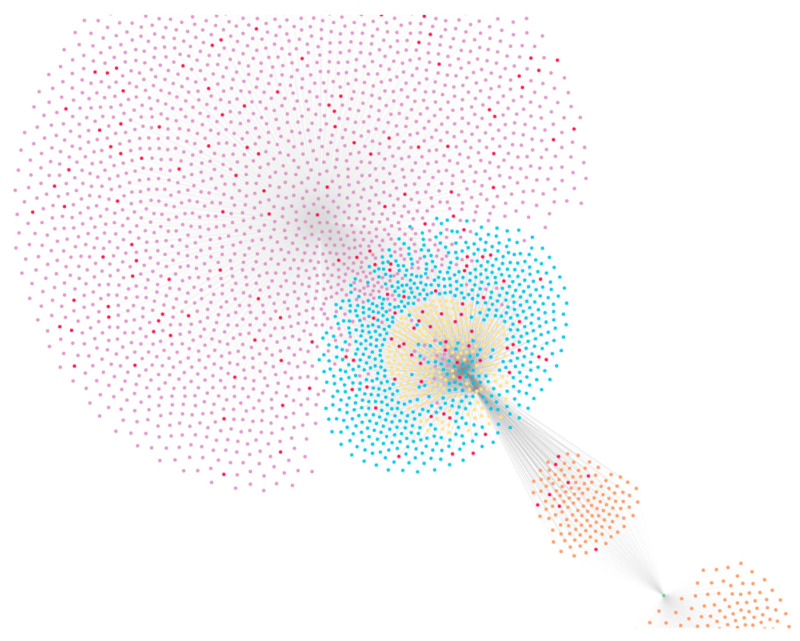
An example of the different communities using the community detection algorithm.

**Figure 5:**
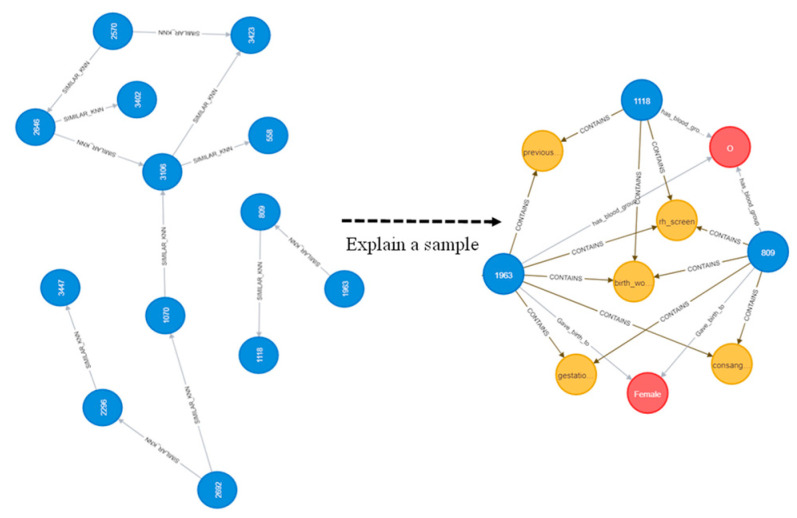
An example of the KNN algorithm showing the reason why patients are similar to each other.

**Figure 6:**
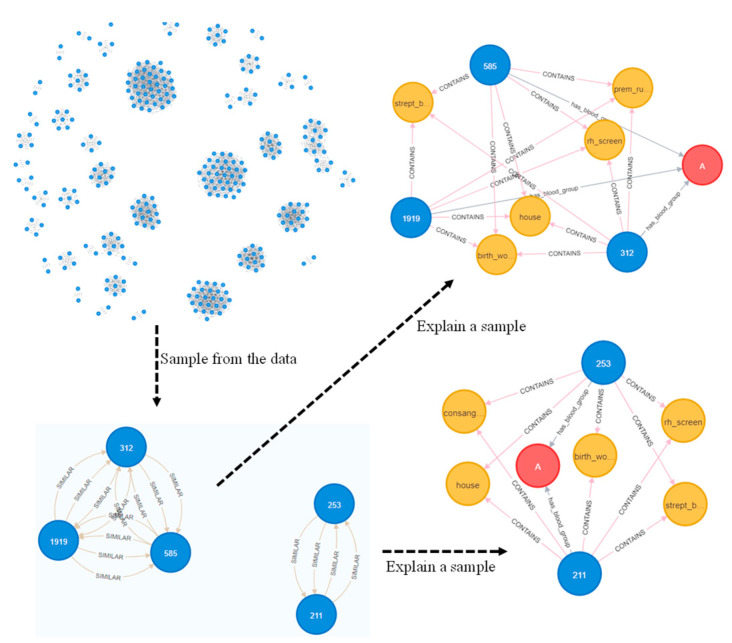
Node similarity algorithm showing the reason why patients may be similar to each other i.e., due to the complications they share. Yellow nodes represent the disease, blue nodes represent the node patients, and red is the blood group.

After creating a knowledge graph, 3884 entities (nodes) were obtained using 25,862 relations by linking the entities. An example graph obtained from the original risk factors using Neo4j is shown in Figure 3. In addition, we have shown the results of using some graph algorithms; for instance, Figure 4 represents multiple communities (sub-graphs) in the dataset using the Louvain algorithm [37]. An example of similar nodes based on the node similarity algorithm is shown in Figure 5, which indicates that patients with similar complications tend to be similar to each other. For instance, patients with patient ID (PID) 1963, 1118, and 809 are similar to each other because they have similar risk factors such as all of them being worried about their upcoming birth, having the same blood group, and having Rh antibodies. In addition, PID 809 and 1963 are having gestational diabetes, consanguinity, etc. Similarly, 1963 and 118 had the previous LBW. Figure 6 also shows the node similarity using KNN models that represent how patients are similar (near) to each other. For instance, it can be seen that patients with PID 585, 1919, and 312 are similar to each other because they share similar complications compared to patients with PID 253 and 211. Furthermore, these two groups of patients are having relatively similar behavior (same blood group, worry about the upcoming birth, Streptococcus B carrier, etc.); therefore, they are close to each other compared to other patient groups. We utilized these data as graph-embedding features. All these combined features were used for classification.

The experimental results obtained using the RF classifier are listed in Table 3. The precision, recall, F-score, AUC-ROC, PR-value for the LBW class, and overall PR-value using the original risk factors were 0.843, 0.887, 0.864, 0.746, 0.306, and 0.878, respectively; when the performance was improved using node embedding, their new values improved to 0.876, 0.886, 0.881, 0.767, 0.330, and 0.886, respectively. The combination of all graph features further improved performance, achieving AUC-ROC and PR-value of 0.777 and 0.355, respectively. Finally, the combination of all features achieved the best performance with precision, recall, F-score, AUC, and PR-value of 0.877, 0.887, 0.882, 0.807, and 0.401, respectively. It can be seen that the best AUC of 0.807 was achieved using a combination of the features while the best PR value of 0.401 was achieved using a combination of graph features. Overall, the performance of the graph features and the combination of features was improved compared to that of the baseline method.

The results obtained using the NB classifier (Table 4) show that node embedding achieved the best performance compared to other features, with precision, recall, F-score, AUC, and PR-value for LBW, and overall PR value of 0.867, 0.889, 0.878, 0.803, and 0.390, and 0.902 respectively. Notably, node embedding improved the AUC by more than 7% compared to the original risk factor; furthermore, the PR value was also increased by approximately 13%.

The results obtained using the LR classifier are shown in Table 5, which shows that the combination of graph features achieved the best classification performance, with precision, recall, F-score, AUC, and PR-value of 0.875, 0.895, 0.885, 0.814, and 0.431, respectively. Similarly, the experimental results obtained using the KNN classifier (Table 6) show that node embedding performed well; however, the overall performance of the KNN classifier was not satisfactory.

The experiments performed using the MLP classifier (Table 7) showed that the best results were achieved using a combination of all the features, achieving AUC and PR-value for LBW class of 0.787 and 0.384, respectively. 

The results obtained using a LightGBM algorithm is shown in Table 8. It can be seen that the best performance was achieved using a combination of all the features which achieved an AUC of 0.819 and PR-value for LBW class of 0.459 for the LBW class. 

Finally, the experiments performed using the XGBoost classifier (Table 9) show that the best performance was achieved using all the (original and graph-based) features which achieved the best performance with precision, recall, F-score, AUC, and PR-value of 0.888, 0.898, 0.893, 0.834 and 0.481, respectively. Furthermore, the performance of other features was also comparable.

## 4. Discussion

LBW is a serious public health concern that poses a serious health challenge to infants. Identifying LBW infants at the earliest stage before birth can help reduce the significant risks associated with the mother and infant. Minimizing the risks associated with LBW in infants can avoid immediate issues such as stunting, low IQ, and even death. Moreover, it can prevent adverse consequences in later life, including obesity, heart disease, diabetes, and other non-communicable diseases. Therefore, in this study, we proposed a promising solution for predicting LBW by using a combination of maternal risk factors and graph-embedding features.

The prevalence of LBW in this study was 11.32%, which is higher than that recently reported by Taha et al. [6]; however, this could be related to differences in study designs, settings, and included participants.

The original tabular dataset was transformed into a knowledge graph and several graph-embedding features were extracted. Different ML classifiers were used to classify LBW infants using various performance metrics. It was demonstrated that the proposed method achieved promising performance. 

Graphs have a unique advantage in that they explore the relationships among patients (Figure 3, Figure 4, Figure 5 and Figure 6) which helps in classification performance. For instance, it can be seen from Figure 5 that patients with ID 809, 1118, and 1963 are similar to each other because they presented the same complications; all of them had birth anxiety and the same blood group. Similarly, the node similarity for the patient in Figure 6 also reveals that patients are close and similar to each other based on the complication type. 

As shown in Table 2, Table 3, Table 4, Table 5 and Table 6, for all ML models, the graph features and combinations of all features achieved better performance compared to the original risk factors. Furthermore, the proposed method achieved a better PR-value, indicating that the model is robust for classifying LBW instances. 

Node embedding features achieved better performance in terms of precision while including node embedding combinations of embedding features, and all feature combinations achieved better AUC (Table 3, Table 4, Table 5, Table 6, Table 7, Table 8 and Table 9). The best AUC of 0.834 was achieved using XGBoost when a combination of all features was used. 

In addition to improved performance, the models developed will be deployed so they can be used by physicians in decision-making. Furthermore, it can be seen in Figure 6 that patients with PID 312, 585, and 1919 share the same complication; therefore, if any patient is at higher risk of a particular disease (LBW, premature rapture membrane, etc.), then the nearby/connected patients may also be at higher risk of such disease. Therefore, the physicians can also closely monitor the patients at risk and propose effective interventions such as early antenatal care, increase physical activity, better nutrition for mothers, and other suggestions to minimize the associated risks. Hence, the proposed knowledge graph-based method can assist physicians with understanding certain patient’s conditions. Therefore, compared to existing techniques, it increases the trust among clinicians to adopt such a method in the clinical setting with confidence.

The dataset used in this study was highly imbalanced; therefore, multiple performance metrics were used. Multiple performance metrics are important to investigate the performance of any method. For instance, it can be seen from Table 6 that the precision, recall, and F-score is high (>80%); however, the AUC and PR-value of the LBW class are low. AUC is calculated by using various threshold values; however, precision and recall are calculated by using one threshold value. Similarly, PR-value shows the tradeoff between recall and precision for different thresholds. Therefore, the small value of AUC and PR-value show that the KNN classifier (Table 6) was unable to distinguish well between LBW and normal BW class. Furthermore, all the classifiers achieved similar performance (except KNN) which shows that the choice of classifier has little impact.

In the proposed work, we have selected an extensive list of 41 important risk factors (Table S1) selected based on the literature [2] and clinical recommendations. However, previous works completed for LBW prediction rely on a small number of maternal risk factors, such as works carried out by Faruk et al. [7], Lu et al. [9], Pollob et al. [11], Do et al. [12], and Lin et al. [13], which used only 9, 14,17, 11, and 21 risk factors, respectively. Furthermore, it can be seen from Table 1 that works completed for LBW prediction need further improvement; for instance, work completed by Faruk et al. [7] and Pollob et al. [11] achieved an AUC of 0.50 and 0.59, while accuracy of only 64% was achieved by Lu et al. [9]. Work carried out by Do et al. [12] and Lin et al. [13] achieved an AUC of 0.845 and 0.72, respectively. Compared to most of the works completed (Table 1), we also achieved a comparatively higher performance with an AUC of 0.834. 

The proposed study provides several advantages such as this using a relatively large cohort study with a large number of important risk factors compared to works completed in the UAE [2,6,14,15]. In addition, to the best of our knowledge, this is the first study that utilized graph embedding features for LBW prediction, especially in the UAE. Although this study has shown significant improvement compared to the original risk factors, it has several limitations. For example, the node embedding and graph features are dependent on the original risk factors and use information from the original dataset. It is challenging to classify a new node that was not included during the graph construction phase, as it may affect the structure of the graph. Embedding features are computationally complex and may require domain knowledge. Moreover, although several ML classifiers were used, no extensive hyperparameter tuning was performed.

In the future, to better evaluate the performance of graph algorithms, we aim to present a unified experimental setup to compare the proposed work with previous works carried out for LBW prediction. Furthermore, since the choice of classifier had little impact on the overall performance, extensive data preprocessing and hyperparameter tuning will be performed for performance improvement. We also aim to include more robust graph algorithms such as graph convolution networks [25,47,48,49,50,51,52]. Furthermore, since we have identified several relevant relationships among the patients (Figure 3, Figure 4 and Figure 5), we aim to address them using a personalized medicine approach.

## 5. Conclusions

In this study, graph-embedding features were incorporated into the original risk factors for LBW classification. Several ML models were used to evaluate classification performance using various performance metrics, which showed that LR achieved the best AUC of 0.834. It was shown that graph embedding features are a promising way to improve performance and can be easily adapted in clinical settings.

## Figures and Tables

**Table 1:** Related works completed for LBW prediction.

References	Method Used	Performance	Limitations
Faruk et al. [7]	LBW prediction using LR, RF. Basic data preprocessing was performed.	AUC of LR was 0.50, Accuracy of RF was 93%.	No other performance other than accuracy was shown for RF. A small set of features were used.
Feng et al. [8]	Fetal weight estimation and classification using ultrasound features. SMOTE [29] was used for data balancing. deep belief network (DBN) for estimation. SVM for classification.	DBN achieved better performance with an MAE of 198.55 g ± 158 g, MAPE of 6.09 ± 5.06%,	LBW and NBW samples were treated as the same class to predict High BW.
Lu et al. [9]	Fetal weight estimation using ensemble (RF, XGBoost, and LightGBM) models.	Accuracy of 64.3% and mean relative error of 7% which was improved by 12% and 3% respectively.	Performance needs further improvement.
Trujillo et al. [10]	Infant BW estimation using support vector regression.	Results show the SVR was able to predict BW with nearly 250 g.	Only one ML model was used for evaluation.
Pollob et al. [11]	LBW classification using LR and decision tree	Sensitivity, specificity, and AUC of 0.99, 0.18, and 0.59 was achieved using LR.	Low performance was achieved with a Specificity of 0.18 and an AUC of only 0.59.
Do et al. [12]	Mortality prediction in very LBW infants using ML (LR, ANN, KNN, RF, SVM) models.	ANN achieved an AUC of 0.845, a sensitivity, and specificity of 0.76 and 0.78, respectively.	A small set of features was used. The sensitivity and specificity need further improvement.
Lin et al. [13]	Prediction of in-hospital length of stay of very LBW infants. Six ML models (KNN, MLP, RF, LR etc) were used.	LR achieved the best performance with AUC of 0.72, precision, recall, and F-score of 0.76, 0.78. 0.744.	Performance needs further improvement.
Khan et al. [2]	BW estimation and LBW classification, SMOTE for data balancing, and multiple sets of features.	LR achieved the best classification performance with an accuracy of 0.90, precision, recall, and F-score of 0.88, 0.90, and 0.89, respectively. Important risk factors were highlighted.	Performance metric such as AUC and PR-value was not used. The classification performance of LBW samples was low.

**Table 2:** Machine Learning models along with its parameter used in this study.

Classifier	Parameter(s)
RF	Batch Size = 100, number of trees = 100, Break Ties Randomly = False, Maximum Depth = None, maximum features = “sqrt”, bootstrap = True, base estimator = DecisionTreeClassifier
SVM	Kernel=Linear, nu=0.5, penalty=l2, loss = squared hinge, maximum iterations = 1000.
Logistic Regression	Batch Size = 100, Ridge = 1.0 × 10^−8^, penalty=l2.
Naïve Bayes	Batch Size = 100, parameters = default.
MLP	Hidden layers = default, activation = relu, alpha = 0.001, learning rate = 0.001, maximum iterations = 200,
KNN	K = 3, distance measure = Euclidean
LightGBM	Batch Size = 100, learning rate = 0.01
XGBoost	Number of estimators = 100, learning rate = 0.01, random state = 42, maximum features = number of features
CatBoost	Iterations = 20, learning rate = 0.01, loss function = cross entropy

**Table 3:** Experiments performed using a random forest classifier.

Method	Precision (SD)	Recall (SD)	F-Score (SD)	AUC (SD)	PR LBW (SD)	PR Overall (SD)
Original	0.843 (0.01)	0.887 (0.001)	0.864 (0.001)	0.746 (0.002)	0.306 (0.001)	0.878 (0.01)
Node Embedding	0.876 (0.05)	0.886 (0.004	0.881 (0.004)	0.767 (0.01)	0.330 (0.01)	0.886 (0.002)
Combination of Graph Features	0.868 (0.01)	0.887 (0.01)	0.877 (0.01)	0.777 (0.01)	0.355 (0.01)	0.888 (0.01)
Combination of all features	0.877 (0.02)	0.887 (0.01)	0.882 (0.01)	0.807 (0.01)	0.401 (0.01)	0.901 (0.01)

**Table 4:** Experiments performed using a naïve Bayes classifier.

Method	Precision (SD)	Recall (SD)	F-Score (SD)	AUC (SD)	PR LBW (SD)	PR Overall (SD)
Original	0.840 (0.01)	0.870 (0.01)	0.855 (0.01)	0.726 (0.01)	0.260 (0.02)	0.868 (0.01)
Node Embedding	0.867 (0.01)	0.889 (0.01)	0.878 (0.01)	0.803 (0.02)	0.390 (0.02)	0.902 (0.01)
Combination of Graph Features	0.855 (0.02)	0.866 (0.01)	0.860 (0.01)	0.779 (0.02)	0.322 (0.01)	0.889 (0.01)
Combination of all features	0.862 (0.01)	0.860 (0.01)	0.861 (0.01)	0.799 (0.02)	0.346 (0.01)	0.895 (0.01)

**Table 5:** Experiments performed using a logistic regression classifier.

Method	Precision (SD)	Recall (SD)	F-Score (SD)	AUC (SD)	PR LBW (SD)	PR Overall (SD)
Original	0.858 (0.003)	0.888 (0.003)	0.873 (0.005)	0.754 (0.003)	0.347 (0.003)	0.884 (0.002)
Node Embedding	0.872 (0.01)	0.895 (0.01)	0.883 (0.01)	0.809 (0.008)	0.419 (0.02)	0.906 (0.004)
Combinations of Graph Features	0.875 (0.01)	0.895 (0.01)	0.885 (0.01)	0.814 (0.01)	0.431 (0.02)	0.908 (0.01)
Combination of all features	0.870 (0.01)	0.884 (0.01)	0.877 (0.01)	0.8189 (0.01)	0.392 (0.01)	0.909 (0.01)

**Table 6:** Experiments performed using a KNN classifier.

Method	Precision (SD)	Recall (SD)	F-Score (SD)	AUC (SD)	PR LBW (SD)	PR Overall (SD)
Original	0.803 (0.01)	0.876 (0.02)	0.838 (0.01)	0.530 (0.02)	0.127 (0.01)	0.806 (0.01)
Node Embedding	0.835 (0.01)	0.873 (0.02)	0.854 (0.01)	0.600 (0.02)	0.166 (0.02)	0.824 (0.01)
Combinations of Graph Features	0.821 (0.01)	0.867 (0.03)	0.843 (0.01)	0.573 (0.03)	0.149 (0.02)	0.817 (0.01)
Combination of all features	0.827 (0.02)	0.876 (0.02)	0.851 (0.01)	0.530 (0.01)	0.132 (0.01)	0.806 (0.01)

**Table 7:** Experiments performed using the MLP Classifier.

Method	Precision (SD)	Recall (SD)	F-Score (SD)	AUC (SD)	PR LBW (SD)	PR Overall (SD)
Original	0.831 (0.01)	0.854 (0.01)	0.842 (0.01)	0.652 (0.02)	0.239 (0.02)	0.850 (0.01)
Node Embedding	0.844 (0.01)	0.860 (0.01)	0.852 (0.01)	0.7217 (0.01)	0.286 (0.02)	0.8734 (0.02)
Combinations of Graph Features	0.848 (0.01)	0.857 (0.01)	0.852 (0.01)	0.745 (0.01)	0.307 (0.01)	0.881 (0.01)
Combination of all features	0.863 (0.01)	0.876 (0.01)	0.869 (0.01)	0.787 (0.01)	0.384 (0.02)	0.897 (0.02)

**Table 8:** Experimental results obtained using the LightGBM Classifier.

Method	Precision (SD)	Recall (SD)	F-Score (SD)	AUC (SD)	PR LBW (SD)	PR Overall (SD)
Original	0.858 (0.01)	0.888 (0.01)	0.873 (0.01)	0.756 (0.01)	0.329 (0.01)	0.882 (0.01)
Node Embedding	0.868 (0.01)	0.911 (0.03)	0.889 (0.01)	0.807 (0.01)	0.409 (0.01)	0.905 (0.01)
Combination of Graph Features	0.872 (0.01)	0.889 (0.01)	0.880 (0.01)	0.811 (0.01)	0.411 (0.01)	0.905 (0.01)
Combination of all features	0.878 (0.01)	0.894 (0.01)	0.886 (0.01)	0.819 (0.02)	0.459 (0.02)	0.913 (0.02)

**Table 9:** Experimental results obtained using the XGBoost Classifier.

Method	Precision (SD)	Recall (SD)	F-Score (SD)	AUC (SD)	PR LBW (SD)	PR Overall (SD)
Original	0.865 (0.01)	0.891 (0.01)	0.878 (0.01)	0.762 (0.01)	0.406 (0.01)	0.891 (0.01)
Node Embedding	0.870 (0.01)	0.892 (0.01)	0.881 (0.01)	0.802 (0.01)	0.410 (0.02)	0.902 (0.01)
Combination of Graph Features	0.872 (0.01)	0.894 (0.01)	0.883 (0.01)	0.822 (0.01)	0.440 (0.01)	0.909 (0.01)
Combination of all features	0.888 (0.01)	0.898 (0.01)	0.893 (0.01)	0.834 (0.01)	0.481 (0.02)	0.916 (0.01)

## Data Availability

The data presented in this study can be made available on request from the Mutaba’ah study. Approval from a research ethics committee may be required.

## Supplementary Materials

The following supporting information can be downloaded at: https://www.mdpi.com/article/10.3390/ijerph20021317/s1, Table S1: Descriptive statistics of the risk factors.
