# Supplementary material for: Infant Low Birth Weight Prediction Using Graph Embedding Features
Source: Int J Environ Res Public Health. 2023 Jan 11;20(2):1317. doi: 10.3390/ijerph20021317 (PMC9859143; doi:10.3390/ijerph20021317)
Supplement: Supplementary file 1 [file ijerph-20-01317-s001.zip › ijerph-2102318-supplementary.pdf]

Infant Low Birth Weight Prediction using Graph Embedding Features

Supplementary Table S.1: Descriptive statistics of the risk factors.

Risk Factors	
	Mean (standard deviation)
Maternal age (years)	31.6 (6.07)
BMI at delivery (kg/m^2)	31.6 (5.96)
First trimester weight (kg)	71.0 (15.66)
Second trimester weight (kg)	74.3 (15.46)
Third trimester weight (kg)	79.4 (14.83)
Height (cm)	158.5 (5.37)
Interpregnancy interval (days)	1014 (782)
	Range
Gravida	1-20
Parity	0-16
Previous caesarian delivery	0-10
Previous pregnancy loss	0-11
	Number (%)
Baby gender	
Male	1,878 (53.6)
Female	1,629 (46.4)
Planned pregnancy	
No	1,514 (46.3)
Yes	1,753 (53.7)
Infertility treatment	
No	2,914 (90.3)
Yes	314 (9.7)
Consanguinity	
No	1,009 (54.7)
Yes	836 (45.3)
Education	
No	2,205 (68.4)
Yes	1,021 (31.6)
Employment	
No	1,647 (51.0)
Yes	1,580 (49.0)
Passive smoking	
No	2,162 (66.0)
Yes	1,114 (34.0)
Physical exercise before pregnancy	
Never	1,683 (55.8)
1-2 times a week	582 (19.3)
3-5 times a week	479 (15.9)

Daily	273 (9.0)
Physical exercise during pregnancy	
Never	1,654 (54.0)
1-2 times a week	722 (23.6)
3-5 times a week	268 (8.7)
Daily	419 (13.7)
House type	
Rent	632 (19.9)
Owned	2,545 (80.1)
Worry about upcoming childbirth	
No	1,024 (32.1)
Yes	2,164 (67.9)
Previous LBW	
No	1,479 (42.1)
Yes	2,030 (57.9)
Previous preterm birth	
No	3,115 (88.8)
Yes	394 (11.2)
Blood group/type	
A	1,048 (29.9)
B	520 (14.8)
AB	138 (3.9)
O	1,803 (51.4)
Preexisting hypertension	
No	3,440 (98.0)
Yes	69 (2.0)
Preexisting diabetes mellitus	
No	3,370 (96.0)
Yes	139 (4.0)
Preeclampsia	
No	3,366 (95.9)
Yes	143 (4.1)
Growth retardation	
No	3,476 (99.1)
Yes	33 (0.9)
Antepartum haemorrhage	
No	3,498 (99.7)
Yes	11 (0.3)
Polyhydramnios	
No	3,427 (97.7)
Yes	82 (2.3)
Oligohydramnios	
No	3,433 (97.8)
Yes	76 (2.2)
Infection of amniotic sac	
No	3,423 (97.6)
Yes	86 (2.4)
Premature rupture of membrane	

No	2,931 (83.5)
Yes	578 (16.5)
Placental disorders	
No	3,477 (99.1)
Yes	32 (0.9)
Placenta previa	
No	3,461 (98.6)
Yes	48 (1.4)
Abruptio placentae	
No	3,438 (98.0)
Yes	71 (2.0)
Streptococcus B carrier	
No	2,620 (74.7)
Yes	889 (25.3)
Genitourinary infection	
No	3,471 (98.9)
Yes	38 (1.1)
Bariatric surgery	
No	3,348 (95.4)
Yes	161 (4.6)
